# Understanding the role of financial capacity in the delivery of opioid use disorder treatment

**DOI:** 10.1186/s12913-023-09179-z

**Published:** 2023-02-16

**Authors:** Erick G. Guerrero, Hortensia Amaro, Yinfei Kong, Tenie Khachikian, Jeanne C. Marsh

**Affiliations:** 1 Research to End Health Disparities Corp, I-Lead Institute, 12300 Wilshire Blvd., Suite 210, Los Angeles, CA 90025 USA; 2 Herbert Werthein College of Medicine and Robert Stempel College of Public Health and Social Work, Florida International University, 11200 SW 8Th St., AHC4, Miami, FL 33199 USA; 3 College of Business and Economics, California State University Fullerton, 800 N. State College Blvd., Fullerton, CA 92831 USA; 4 Crown Family School of Social Work, Policy, and Practice, University of Chicago, 969 E. 60Th St., Chicago, IL 60637 USA

**Keywords:** Financial capacity, Medicaid, Access, Engagement, Opioid use disorder treatment

## Abstract

Opioid treatment programs must have adequate financial capacity to sustain operations and deliver a high standard of care for individuals suffering from opioid use disorder. However, there is limited consistency in the health services literature about the concept and relationship of organizational financial capacity and key outcome measures (wait time and retention). In this study, we explored five common measures of financial capacity that can be applied to opioid treatment programs: (a) reserve ratio, (b) equity ratio, (c) markup, (d) revenue growth, and (e) earned revenue. We used these measures to compare financial capacity among 135 opioid treatment programs across four data collection points: 2011 (66 programs), 2013 (77 programs), 2015 (75 programs), and 2017 (69 programs). We examined the relationship between financial capacity and wait time and retention. Findings from the literature review show inconsistencies in the definition and application of concepts associated with financial capacity across business and social service delivery fields. The analysis shows significant differences in components of financial capacity across years. We observed an increase in average earned revenue and markup in 2017 compared to prior years. The interaction between minorities and markup was significantly associated with higher likelihood of waiting (IRR = 1.077, *p* < .05). Earned revenue (IRR = 0.225, *p* < .05) was related to shorter wait time in treatment. The interaction between minorities and equity ratio is also significantly associated with retention (IRR = 0.796, *p* < .05). Our study offers a baseline view of the role of financial capacity in opioid treatment and suggests a framework to determine its effect on client-centered outcomes.

## Introduction

The current substance use disorder (SUD) treatment system has struggled to meet the service needs of an increasingly diverse population [26, 47], particularly those suffering from opioid use disorder (OUD; [25, 30]. Because many SUD treatment programs are small and financially unstable, they face high risks of discontinuing services or reducing the quality of care [23, 33]. Furthermore, low revenues and net assets are often reported as barriers to financing comprehensive services like mental health and social services [31, 43, 47]. Delivering these services is associated with improved SUD treatment outcomes [2, 3, 39]. To effectively utilize recent increases in funding for OUD treatment services in the United States from pharmaceutical settlements [28] and the Biden administration [7], it is critical to understand how financial capacity is measured and relates to program outcomes in the OUD treatment system.

Financial capacity, defined as programs’ revenue- and profit-generating resources to sustain treatment services, is a key factor in delivering a high standard of care [32]. Programs with more resources can provide better quality of care, as measured by offering comprehensive services and reporting greater initiation and engagement rates [20–22, 26, 27]. However, current understanding of financial capacity and its relevant indicators in the context of OUD treatment systems is limited. In this paper, we draw from the financial literature across service industries to operationalize the financial capacity of OUD treatment organizations based on the most common capacity indicators: (a) reserve ratio, (b) equity ratio, (c) markup, (d) revenue growth, and (e) earned revenue.

Research on social service organizations has suggested that operating reserves, defined as unrestricted net assets, form an important part of an organization’s overall financial health [6, 18]. Unrestricted net assets represent assets the organization can fully control, whereas temporarily and permanently restricted net assets have conditions that limit their use. The monthly reserve ratio builds on this concept and represents the unrestricted net assets that an organization can use to replace lost revenue to continue operations—i.e., reserves can serve as a financial cushion. The reserve ratio also represents the resources organizations must have to start new programs, expand existing ones, or invest in infrastructure [6, 18].

The equity ratio represents the proportion of net assets that an organization owns, “free and clear” of liabilities. Values greater than 0.50 and closer to 1 mean the organization’s assets are more unencumbered by liabilities, whereas values less than 0.50 and closer to 0 mean a greater proportion of assets are offset by liabilities. The ideal equity ratio largely depends on an organization’s industry and its goals; e.g., a bank is likely to have a lower equity ratio, meaning it has a higher proportion of debt, compared to a technology company [16]. In health care organizations, inability to move into solvency (wherein assets exceed liabilities) increases the risk that the organization will close [33, 46].

Markup is defined as unrestricted net assets from the current year minus unrestricted net assets of the prior year plus depreciation expenses, divided by total expenses. Markup is closely related to the organization’s profit margin, which is the annual rate at which it builds its unrestricted net assets—i.e., the margin of revenue left over after accounting for operating costs. Higher markup can lead to greater profitability, thus reducing a health care program’s risk of closing [33]. However, high markups of the price for health care services may reduce access to care for clients with less financial resources [41, 45, 48] without necessarily leading to a higher quality of care [4, 17, 50].

Revenue growth, defined as the rate of new revenue generation, is critical to sustaining long-term operations [33]. Annual revenue growth is the total revenue of the current year minus the total revenue of the prior year divided by the total revenue of the prior year. A positive growth rate of 3% or more is generally advisable [9]. The literature on whether diversifying revenue sources encourages revenue growth has shown mixed findings [10, 15], though limited evidence suggests that revenue growth may be improved by concentrating on revenue sources in one area (e.g., governmental funds but diversifying in that area (e.g., obtaining funds from different levels of government; [11]. Policy often affects revenue growth by restricting or adding certain forms of revenue generation. For example, the Affordable Care Act’s expansion of public health insurance coverage led to higher revenue growth for health care organizations [44, 49].

Another critical indicator of financial capacity is earned revenue, or the proportion of revenue earned from providing products or services (i.e., revenue from reimbursements, service-based contracts, fees), as opposed to revenue from other sources (e.g., donations, investments). It roughly indicates the degree to which the organization is self-sufficient and able to generate its own revenue versus depending on external donations and grants. Earned revenue can serve as an important independent financial resource and thus, can be an indicator of sustainable program operation [12, 13]. Limited evidence from research on nonprofits suggests that higher proportions of earned revenue from sources that align with an organization’s mission positively affect the organization’s outcomes [12, 13].

### Influence of Medicaid expansion on financial capacity

Because Medicaid is the largest funder of OUD treatment services in the United States, Medicaid expansion has provided one of the largest boosts to OUD programs’ financial capacities and the potential to enhance treatment access and engagement [1, 20, 21, 26, 37, 42].

In general, Medicaid expansion is associated with better financial performance among health care organizations in terms of greater decreases in uncompensated care, increases in average annual Medicaid-generated revenue, a better equity ratio, fewer closures, and better operating margins [5, 32, 34, 35]. The expansion of California’s Medicaid program (Medi-Cal) led to a 95% increase in patient revenue for Medi-Cal at city and county hospitals from 2013 to 2019, reaching $6.9 billion by 2019 [14]. Meanwhile, primary care net revenue from Medi-Cal more than doubled, growing from $2.1 billion in 2013 to $4.3 billion in 2019 [14]. Data from OUD treatment programs show that compared to programs in nonexpansion states, programs in expansion states were more likely to offer a wider range of OUD treatment options and services [29, 40, 51], which may reflect Medicaid-induced improvements in financial capacity to provide these services. National data on SUD spending show that Medi-Cal paid for 21% ($5.4 billion) of the $24 billion spent on SUD treatment in 2009 and is estimated to be responsible for a 38% ($6.8 billion) increase in spending on SUD treatment between 2009 and 2020 [37].

In sum, establishing consistency in the conceptualization, definition, and application of indicators of financial capacity in health care requires deeper examination, particularly during a period of Medicaid expansion. Because this system is tasked with enhancing access to quality care in response to the insidious OUD epidemic, it is critical for the OUD treatment system to integrate understanding of how to apply financial capacity measures to sustain a high national standard of OUD care. In the present study, we conducted exploratory comparative analyses to understand variation across measures of financial capacity (e.g., revenues, net assets, and cash flow) in a large, diverse public OUD treatment system across several years both before and after Medicaid expansion. Additionally, we explored the relationship between financial capacity (e.g., revenues, net assets, and cash flow) and indicators of access and retention using racial disparities as mediating factors.

## Methods

### Data and sample

We relied on client administrative data from the Los Angeles County Participant Reporting System and Integrated Substance Abuse Treatment to End Disparities Program Survey dataset [20–22, 26, 25]. The data came from a study funded by the National Institute on Drug Abuse (R01DA048176). As detailed elsewhere [38], this study merged four waves of administrative client records with OUD treatment program surveys (2011, 2013, 2015, and 2017).

### Dependent variables

The first dependent variable in this study was wait time, a measure of treatment access defined as the number of days clients spent on a waiting list before being admitted to a treatment program. The second variable was retention, defined as number of days clients spent in treatment (i.e., treatment duration in days). These outcomes are measured at the client level and have been used in several studies across datasets, time periods, and treatment systems [8, 19, 36, 38].

### Independent variables

The independent variables in our model consisted of five financial capacity variables—reserve ratio, equity ratio, markup, revenue growth, earned revenue—and client and program characteristics.

The five financial capacity measures are at the program level and obtained from publicly available records from the Internal Revenue Service. These measures were operationalized as follows: Reserve ratio was calculated as unrestricted net assets minus net assets divided by annual expenses divided by 12. Equity ratio was calculated as assets minus liabilities divided by total assets. Markup was computed as unrestricted net assets of current year minus unrestricted net assets of prior year plus depreciation expenses divided by total expenses. Revenue growth was equal to the total revenue of the current year minus the total revenue of the prior year divided by the total revenue of the prior year. Earned revenue was equal to program service revenue divided by total revenue.

Client characteristics included sex (0 = *male*, 1 = *female*); year (1 = *2011*, 2 = *2013*, 3 = *2015*, 4 = *2017*); age (coded in years as a continuous variable); race and ethnicity (1 = *White*, 1 = *African American, Latino, or other*); education (years in school), employment (0 = *unemployed*, 1 = *employed*); homelessness (0 = *no*, 1 = *yes*; based on counselor assessment); mental illness (0 = *no*, 1 = *yes*; based on client report of diagnosis of mental illness); age at onset of primary drug use (years); days using primary drug (number of days of primary substance use during 30 days prior to admission); number of children younger than 18 living at home (continuous variable); eligibility for Medi-Cal (California’s Medicaid program in California; 0 = *no*, 1 = *yes*); number of prior SUD treatment episodes (any alcohol or drug treatment or recovery program); methadone treatment (receiving methadone treatment from OUD treatment program; 0 = *no*, 1 = *yes*). Medi-Cal payment acceptance is measured at the program level (program accepts Medi-Cal; 0 = *no*, 1 = *yes*).

### Data analysis

To conduct the comparative trend analysis of all variables by year, we used analysis of variance and chi-square analysis. We explored changes over time in client characteristics such as age, race, education, employment, homelessness status, mental health issues, and Medi-Cal eligibility and program characteristics such as Medicaid acceptance. To analyze the relationship between financial capacity and outcomes of wait time and retention with racial disparities as a moderator, we conducted multilevel negative binomial regression, reporting differences in terms of incident rate ratios (IRRs). IRRs greater than 1 reflect higher financial capacity indicators and higher proportions of minorities and women in treatment; IRRs less than 1 reflect lower financial capacity indicators and lower proportions of minorities and women in treatment.

## Results

### Comparative analysis of client characteristics, program characteristics, and financial capacity by year

**Fig. 1:**
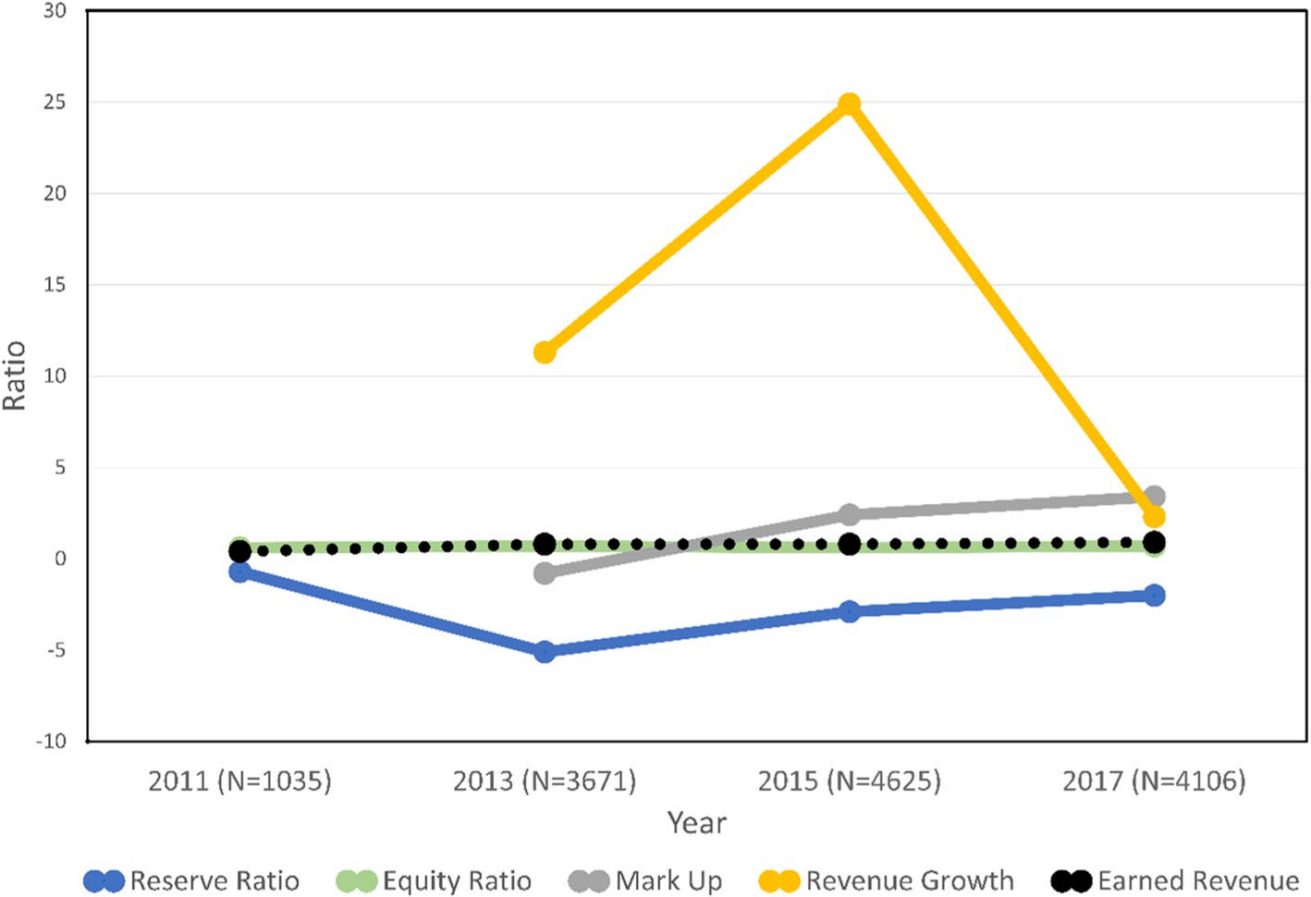
Trends in Financial Capacity Among Opioid Treatment Programs

The comparative analysis of client, program, and financial capacity variables by year is presented in Table 1 and Fig. 1. Acceptance of Medicaid, the largest payer of SUD treatment services, increased across years until reaching 100% of programs accepting Medicaid payments by 2017 (*p* < 0.001). The percentage of female clients decreased over time (*p* < 0.01). Individuals who self-identified as White or Latino increased, whereas those identifying as African American decreased over time (*p* < 0.001). Most significantly, individuals experiencing homelessness increased over time, and those eligible for Medicaid more than doubled from 2011 to 2017 (*p* < 0.001).

**Table 1:** Comparative analysis of financial capacity variables by year

	2011	2013	2015	2017
	(*n* = 1,035)	(*n* = 3,671)	(*n* = 4,625)	(*n* = 4,106)
*Program characteristics*				
Medicaid acceptance**	914 (88.7%)	3,332 (93.7%)	4,444 (97.5%)	3,885 (100.0%)
Reserve ratio**	-0.7 (2.3)	-5.1 (3.7)	-2.9 (1.8)	-2.0 (4.2)
Equity ratio**	0.6 (0.2)	0.7 (0.7)	0.6 (0.2)	0.7 (0.6)
Mark up**	–	-0.8 (5.0)	2.4 (5.6)	3.4 (7.1)
Revenue growth**	–	11.3 (14.1)	24.9 (30.7)	2.3 (46.9)
Earned revenue**	0.4 (0.4)	0.8 (0.3)	0.8 (0.2)	0.9 (0.3)
*Client characteristics*				
Female*	358 (34.6%)	1,074 (29.3%)	1,406 (30.4%)	1,290 (31.4%)
Age**	41.8 (13.7)	43.1 (13.3)	41.1 (13.1)	41.9 (13.5)
Race**				
White	445 (43.0%)	1,458 (39.7%)	2,137 (46.7%)	1,931 (47.8%)
Black	173 (16.7%)	379 (10.3%)	407 (8.9%)	373 (9.2%)
Latino	373 (36.0%)	1,664 (45.3%)	1,838 (40.1%)	1,583 (39.2%)
Other	44 (4.3%)	170 (4.6%)	197 (4.3%)	154 (3.8%)
Education (years)**	11.5 (2.7)	11.4 (2.7)	11.6 (2.7)	11.3 (3.2)
Employed**	108 (10.4%)	760 (20.7%)	969 (21.0%)	844 (20.6%)
Homeless status**	149 (14.4%)	453 (12.3%)	580 (12.5%)	637 (15.5%)
Mental health issues**	276 (26.7%)	756 (20.6%)	1,002 (21.7%)	905 (22.0%)
Medi-Cal eligible**	453 (43.8%)	1,176 (32.0%)	3,093 (66.9%)	2,956 (72.0%)

^*^p < .01. **p < .001

In terms of financial capacity factors, all measures statistically differed across years (*p* < 0.001). Reserve ratios fluctuated greatly, with a tendency toward lower reserve ratios in 2017 compared to 2011. Equity ratios only showed slight variation of less than 10% across the study period. In sharp contrast, markup increased exponentially, growing more than 3 times from 2011 to 2017 (*p* < 0.001). Revenue growth was based on data from the current and previous year, starting in 2013. Revenue growth doubled from 2013 to 2015, then decreased to less than a quarter in 2017. In contrast, earned revenue doubled from 2011 to 2013, then increased slightly from 2013 to 2017. In sum, each indicator of financial capacity showed a different trend. Revenue growth and earned income growth were consistent with improved financial performance identified in the expansion of Medicaid-related revenues among OUD treatment programs and other health care organizations.

### Association of client characteristics, program characteristics, and financial capacity and outcomes of wait time and retention with racial disparities as a mediator

The multilevel negative binomial regression models in Table 2 show that the client and program characteristics significantly associated with wait time and retention were consistent with previous studies [19–21, 25, 38]. Specifically, being female (IRR = 1.672, *p* < 0.01), being homeless (IRR = 1.836, *p* < 0.01), having mental health issues (IRR = 1.773, *p* < 0.001), days using primary drug (IRR = 1.051, *p* < 0.001), court-mandated referral (IRR = 3.815, *p* < 0.01), residing with children younger than 18 (IRR = 1.251, *p* < 0.05), and number of prior treatment episodes (IRR = 1.090, *p* < 0.05) were related to longer wait time in treatment. Older clients (IRR = 1.004, *p* < 0.05) and those with prior treatment episodes, a measure of severity (IRR = 1.022, *p* < 0.001), were more likely to remain in treatment longer.

**Table 2:** Negative binomial models for wait time and retention

	Wait time		Retention	
	IRR	95% CI	IRR	95% CI
*Client characteristics*				
Female	1.672***	1.254, 2.230	1.030	0.954, 1.112
Year	0.639	0.307, 1.328	1.113	0.998, 1.241
Age	1.009	0.993, 1.024	1.004*	1.001, 1.007
Minorities (Black, Latino, Other)	1.335	0.733, 2.431	0.941	0.774, 1.144
Education (years)	1.062	0.990, 1.139	0.998	0.989, 1.006
Employed	1.266	0.820, 1.955	1.072	0.971, 1.183
Homeless	1.836**	1.251, 2.696	0.877*	0.792, 0.970
Mental health issues	1.773***	1.265, 2.485	1.011	0.936, 1.091
Age using primary drug	0.999	0.976, 1.024	0.994***	0.991, 0.997
Days using primary drug	1.051***	1.026, 1.076	0.989***	0.984, 0.994
Court mandated referral	3.815**	1.473, 9.880	0.860	0.729, 1.015
# Children under 18	1.251*	1.048, 1.493	0.961***	0.938, 0.985
Medi-Cal eligible	0.225***	0.109, 0.465	1.074	0.981, 1.176
# prior episodes	1.090*	1.016, 1.168	1.022***	1.014, 1.030
Medication for opioids	0.636	0.078, 5.180	1.178	0.913, 1.519
*Program characteristics*				
Medi-Cal payment acceptance	0.794	0.199, 3.172	0.763	0.558, 1.043
*Financial variables*				
Reserve ratio	1.098	0.714, 1.688	1.013	0.993, 1.033
Equity ratio	0.946	0.355, 2.523	1.493***	1.168, 1.908
Mark up	1.015	0.950, 1.084	0.993*	0.986, 0.999
Revenue growth	1.000	0.998, 1.001	1.000	1.000, 1.000
Earned revenue	0.225*	0.053, 0.955	0.745*	0.559, 0.994
*Interactions*				
Minorities*Equity ratio			0.796*	0.645, 0.981
Minorities*Markup	1.077*	1.014, 1.143		
Minorities*Earned revenue			1.256*	1.035, 1.525
log Alpha	3.884	3.452, 4.317	0.239	0.134, 0.343
# treatment episodes		5028	5028	
# treatment programs		70	70	

*IRR* Incidence rate ratio, *CI* Confidence interval

^*^p < 0.05, **p < 0.01, ***p < 0.001

The analysis of the relation of financial capacity variables to wait time and retention showed significant main effects. Earned revenue was related to lower wait time (IRR = 0.225, *p* < 0.05) and shorter retention (IRR = 0.745, *p* < 0.05). Markup also was related to shorter retention (IRR = 0.993, *p* < 0.05), whereas equity ratio was related to longer retention (IRR = 1.499, *p* < 0.05). The analysis of racial composition of treatment programs showed the interaction between minorities and earned revenue was related to longer retention (IRR = 1.256, *p* < 0.05), whereas the interaction between minorities and markup was significantly associated with higher likelihood of waiting (IRR = 1.077, *p* < 0.05). The interaction of minorities and equity ratio (IRR = 0.796, *p* < 0.05) was related to shorter retention in treatment. Overall, the financial capacity variables that increased during the period of Medicaid expansion, earned revenue, markup, and revenue growth, were related to the outcome variables of wait time and retention. Further, the proportion of minorities in a treatment program served to moderate, or change, the strength and direction of these relationship.

## Discussion

In this study, we described the definitions and applications of financial capacity concepts; explored how measures of these concepts varied across years during Medicaid expansion; and examined the relation of these measures to wait time and retention in one of the largest and most diverse OUD treatment systems in the United States. Our comparative trend analysis of financial capacity measures from the OUD treatment system in Los Angeles County showed distinct patterns for each measure. Although both earned revenue and markup were higher in 2017 compared to prior years, each measure followed a different pattern and growth rate over years. The literature has suggested that higher revenue and markup are associated with greater stability to sustain the delivery of services [33, 44]. This is encouraging news for a system that requires meaningful investment to respond to the unmet service needs of the nation, particularly underserved minority communities [24]. In contrast, equity ratio and reserve ratio varied significantly, and results suggest a system with inconsistent support.

Our analysis of the relation of financial capacity measures to outcomes showed the financial capacity variables that increased during the period of Medicaid expansion, earned revenue, markup, and revenue growth, were related to the outcome variables of wait time and retention. Earned revenue was associated with lower odds of wait time, but also with lower odds of retention, contrary to our assumptions. Markup was also associated with lower odds of retention. Several factors may contribute to a higher rate of retention. Higher revenues and markup do not seem to directly contribute to retention, but revenue played a role in enhancing access to opioid treatment.

Indicators of financial capacity had the most significant associations in diverse programs compared with programs serving mostly White clients. Finding show that when programs had more clients from minority backgrounds (Blacks and Latinos), markup predicted higher odds of wait time and equity ratio predicted lower odds of retention, contrary to our assumptions. The most significant finding suggests that programs serving more minority clients and reporting higher earned revenue were better able to retain these clients longer. Such “minority programs” are generally smaller and have fewer resources and professionals with graduate degrees compared with programs serving mostly White clients [20–22].

Our findings should be considered in light of the limitations of the study. Our administrative, health service and financial data are unique and represent one of the largest OUD treatment systems in the nation. However, these data have several limitations related to measurements and statistical approaches to explore their relationship with outcomes. Financial capacity data was collected from non-profit treatment organization reporting to the Internal Revenue Service. As such, the accuracy of financial measures was not validated by a third party. Second, to respond to collinearity issues due to the high number of measures considered in statistical models, we had to transform some measures and adjust their interpretation. For instance, we treated year as a continuous variable in the negative binomial regression model. Third, we ran multilevel models because the 5,028 treatment episodes (client characteristics) were embedded within 70 treatment programs (program characteristics). All client characteristic variables were in level 1 while all program characteristics and financial variables are in level 2. This is represented in Table 2. Fourth, to present our findings in a coherent manner, we only presented interactions that were statistically significant. We also protected our models from the risk of collinearity by not including the interactions of all financial variables with minorities. We tested interactions one by one and then combined the significant ones. Despite these limitations and considering the limited datasets with epidemiological, health services and financial measures in addiction treatment, this study offers an important baseline for health services research.

Overall, our finding that minority programs reporting higher revenues retain minority clients better compared with their counterparts is critical because this is the first study to identify revenue increase as a strategy to reduce disparities in treatment retention. This finding has significant implications for how the opioid treatment system structures and regulates the financing of OUD treatment for the benefit of vulnerable populations.

This is one of the first studies to examine the role of financial capacity in a large OUD treatment system and further, to propose definitions for key financial capacity measures. As the nation faces increasing demand for effective OUD treatment, it is necessary to establish definitions, measures, and applications of financial capacity variables to understand, assess, and determine the impact of financial indicators on treatment process and quality of care. Because federal and state government leaders seek to inject significant financial resources into the OUD treatment system, it is critical to identify the role of key indicators of program financial capacity to improve client-centered outcomes.

## Data Availability

The datasets used and/or analyzed during the current study are available from the corresponding author and with permission of the Los Angeles County Department of Public Health on reasonable request.

## Abbreviations

SUD: Substance use disorder
OUD: Opioid use disorder
Medi-Cal: California’s Medicaid program
